# A review for the pharmacological effects of paeoniflorin in the nervous system

**DOI:** 10.3389/fphar.2022.898955

**Published:** 2022-08-15

**Authors:** Hongxiang Hong, Xu Lu, Chunshuai Wu, Jiajia Chen, Chu Chen, Jinlong Zhang, Chao Huang, Zhiming Cui

**Affiliations:** ^1^ Department of Spine Surgery, The Second Affiliated Hospital of Nantong University, Nantong, Jiangsu, China; ^2^ Department of Pharmacology, School of Pharmacy, Nantong University, Nantong, Jiangsu, China

**Keywords:** paeoniflorin, brain, neuroinflammation, oxidative stress, neuroprotection

## Abstract

Paeoniflorin, a terpenoid glycoside compound extracted from Paeonia lactiflora Pall, shows preventive and therapeutic effects in various types of nervous system disorders. However, to date, no comprehensive knowledge on the pharmacological effects of paeoniflorin on the nervous system is available online. Clarification of this issue may be useful for the development of paeoniflorin as a new drug for the treatment of nervous system disorders. To this end, the authors summarize the pharmacological aspects of paeoniflorin and its possible mechanisms, such as restoration of mitochondrial function; inhibition of neuroinflammation, oxidative stress, and cellular apoptosis; activation of adenosine A1 receptor, cAMP response element-binding protein (*CREB*) and extracellular signal-regulated kinase 1/2 (ERK1/2); or enhancement of brain-derived neurotrophic factor and serotonin function, in the prevention of disorders such as cerebral ischemia, subarachnoid hemorrhage, vascular dementia, Alzheimer’s disease, Parkinson’s disease, depression, post-traumatic syndrome disorder, and epilepsy, by reviewing the previously published literature.

## Introduction

**FIGURE 1:**
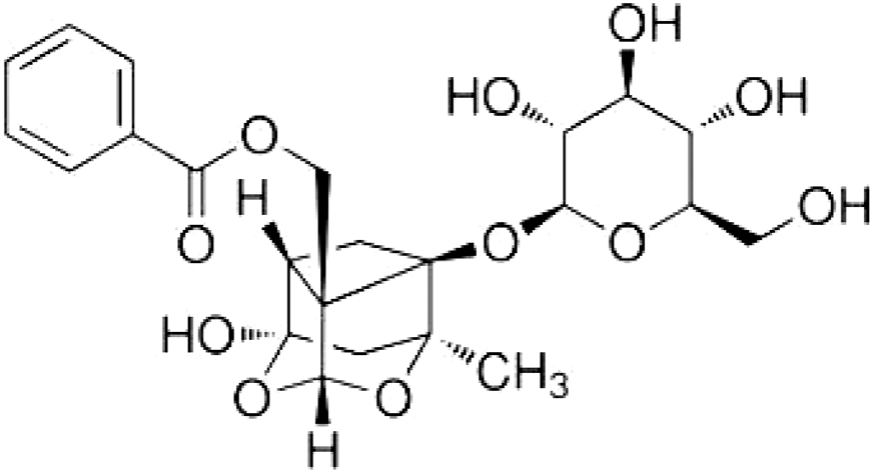
The structure of paeoniflorin.

Paeoniflorin (Figure 1; molecular formula: C_23_H_28_O_11_; molecular weight: 482.46; solvent: dimethyl sulfoxide) is a highly water-soluble single terpenoid glycoside compound first extracted from Paeonia lactiflora Pall (Liu et al., 2005a). It is also found in other Paeoniaceae families, such as Paeoniae veitchii and Paeoniae suffrusticosa (Zhang et al., 2019). Functional studies have shown that paeoniflorin administration exhibits various pharmacological activities, such as anti-inflammatory (Zhang et al., 2019; Xin et al., 2019), anti-oxidative (Yuan et al., 2020; Wang et al., 2020), anti-allergic (Wang and Cheng, 2018), anti-proliferative (Xiang et al., 2020), and anti-diabetic (Sun et al., 2021a) activities, making it a promising agent for ameliorating numerous pathological processes, such as allergy (Wang and Cheng, 2018), cancer (Xiang et al., 2020), and diabetes-associated complications (Sun et al., 2021a). In the nervous system, paeoniflorin administration shows a variety of pharmacological activities, such as ameliorating the pathogenesis of cerebral ischemia (Wu et al., 2020a, Guo et al., 2012, Liu et al., 2005b, Xiao et al., 2005), subarachnoid hemorrhage (SAH) (Wang et al., 2020), learning and memory deficits (Sun et al., 2017), vascular dementia (Zhang et al., 2016; Luo et al., 2018), Alzheimer’s disease (Gu et al., 2016; Kong et al., 2020), Parkinson’s disease (Liu et al., 2006; Zheng et al., 2017), depression (Chen et al., 2019; Li et al., 2020), and post-traumatic syndrome disorder (PTSD) (Chen et al., 2020a), pain (Zhou et al., 2019; Liu et al., 2020), and glioblastoma (Wang et al., 2018a; Yu et al., 2019a), suggesting that dietary supplementation with paeoniflorin may have obvious neuroprotective effects on the central nervous system. A comprehensive understanding of the pharmacological effects of paeoniflorin on the central nervous system, which is not available in the literature published online, may advance the development of paeoniflorin as a new drug for the treatment of central nervous system disorders.

Pharmacodynamic studies had shown that paeoniflorin has a low oral bioavailability (about 2.32%) (Yu et al., 2019b), which is due to its metabolic conversion to benzoic acid by carboxylesterase in the intestinal microbiota (Takeda et al., 1995; Yu et al., 2019b). Pharmacological inhibition of carboxylesterase activity can block the conversion of paeoniflorin to benzoic acid in the intestinal microbiota and in the paeoniflorin co-incubation system (Yu et al., 2019b). This property indicates that the paeoniflorin may have difficulty reaching the brain. However, previous studies have also reported that paeoniflorin can be detected in the hippocampus when administered subcutaneously (Xia et al., 2007), and intravenous administration of paeoniflorin to male rats can result in a detectable paeoniflorin in the brain (He et al., 2004). Paeoniflorin has also been detected in cerebral nuclei of the amygdala, hypothalamus, thalamus, and cortex 30 min after intraperitoneal administration (Zhang et al., 2008). Once paeoniflorin enters the brain, it produces neuroprotective effects through a variety of mechanisms, including inhibition of neuroinflammation (Liu et al., 2006; Guo et al., 2012; Liu et al., 2020), oxidative stress (Lan et al., 2013; Wang et al., 2018b; Wang et al., 2020), cellular apoptosis (Gu et al., 2016; Cong et al., 2019; Wang et al., 2020), and mitochondrial dysfunction (Li et al., 2014; Wang et al., 2014; Cong et al., 2019), activation of cyclic adenosine monophosphate-response element binding protein (*CREB*) (Zhang et al., 2017; Hu et al., 2019) and protein kinase B (Akt) (Wu et al., 2013; Ma et al., 2018), inhibition of c-Jun N-terminal kinase (JNK) (Chu et al., 2017), or enhancement of brain-derived neurotrophic factor (BDNF) (Chen et al., 2019; Mu et al., 2020) and serotonin functions (Qiu et al., 2013; Qiu et al., 2018). Another possible mechanism for the neuroprotective effect of paeoniflorin is its conversion to benzoic acid, an intermittent compound that can be transported to the brain via monocarboxylate transporter 1 (MCT1) (Kido et al., 2000) and produce neuroprotective effects in schizophrenia (Lane et al., 2013), dementia (Lin et al., 2019; Lin et al., 2021), and early-phase Alzheimer’s disease (Lin et al., 2014). The results on the distribution of paeoniflorin in the brain suggest that paeoniflorin may be able to directly cross the blood-brain barrier, and that an important way by which paeoniflorin exerts neuroprotective effects in the central nervous system may be related to its direct action on cells in the brain.

Although paeoniflorin can enter the brain when injected subcutaneously (Xia et al., 2007), intravenously (He et al., 2004), or intraperitoneally (Zhang et al., 2008), researchers still do not know exactly how paeoniflorin crosses the blood-brain barrier. Improving the delivery of paeoniflorin to the brain could improve its therapeutic efficacy in nervous system disorders. To this end, future studies should focus on the physical, chemical, and structural chemistry, biocompatibility, pharmacology, and mechanisms of paeoniflorin to cross the blood-brain barrier. At this stage, several methods could be considered to improve the efficacy of paeoniflorin when delivered to the brain. First, based on the possible promoting effect of the main components of Chuanxiong (a messenger drug that can increase the distribution of drugs in brain tissues) (Wang et al., 2011; Zhen et al., 2012), such as ligustilide, senkyunolide I and senkyunolide A, on the ability of paeoniflorin to cross the blood-brain barrier under *in vitro* conditions (Hu et al., 2016), researchers could use Chuanxiong or its components to improve the efficacy of paeoniflorin delivery to the brain. Second, researchers could use the other alternative methods to improve the delivery efficacy of paeoniflorin to the brain. Intranasal administration is an effective method to reach the brain directly through the olfactory and trigeminal neural systems, bypassing the blood-brain barrier. In a previous study, researchers found that there is a high concentration of paeoniflorin in the central nervous after intranasal administration of the nanocrystal formulation of paeoniflorin (Wu et al., 2020b), suggesting that intranasal administration may be an alternative method to improve the efficacy of paeoniflorin delivery to the brain. Third, because paeoniflorin can be converted to benzoic acid in advance, researchers could use the strategy of carboxylesterase inhibition to block the conversion of paeoniflorin to benzoic acid (Yu et al., 2019b) and thus increase the concentration of paeoniflorin in the brain. These hypotheses should be tested in future studies.

It is worth noting that although there are few human clinical experiments on the pharmacological effects of paeoniflorin in diseases of the nervous system, in recent years clinical studies have been conducted on the treatment of cerebrovascular diseases including cerebral infarction, cerebral hypoperfusion syndrome and cerebral ischemia, and intracranial arterial stenosis of the brain by Chinese recipes containing Paeonia, such as the Bu Yang Huan Wu, Si Wu, and Huang Chi Wu decoctions. The detailed information on these studies can be found in the following websites: http://www.chinadrugtrials.org.cn and http://www.chictr.org.cn (the Chinese Clinical Trial Registry), http://clinicaltrials.gov (the Global Clinical Trial Registry), and http://clinicaltrialsregister.eu (the EU Clinical Trials Register). Therefore, further elucidation of the pharmacological aspects of paeoniflorin in the nervous system could be of great significance for the development of paeoniflorin as a novel drug for the treatment of central nervous system disorders. However, no comprehensive review on this topic has been presented in public sources so far. In this context, the authors summarize and discuss the pharmacological effects of paeoniflorin and its possible mechanisms in the prevention and/or treatment of nervous system disorders by searching previously published literatures and databases, with the aim of advancing the application of paeoniflorin and paeoniflorin-containing drugs in clinical use and daily life.

## Pharmacological effects of paeoniflorin in cerebral ischemia

**FIGURE 2:**
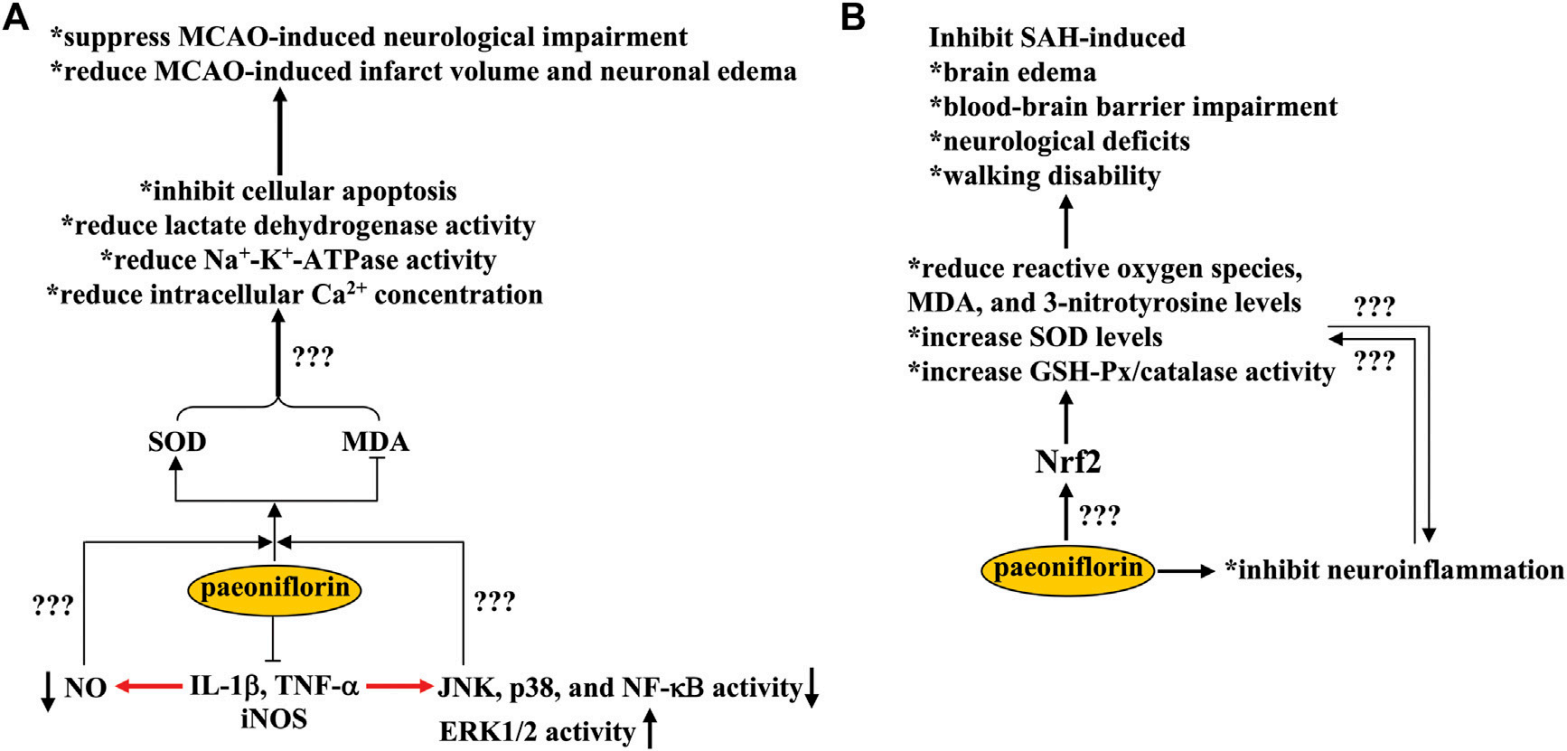
Effects and mechanisms of paeoniflorin in cerebral ischemia and subarachnoid hemorrhage. **(A)** Paeoniflorin administration suppresses MCAO-induced neurological impairment and reduces MCAO-induced infarct volume and neuronal edema through mechanisms dependent on SOD elevation and MDA decrease, including inhibition of cellular apoptosis, lactate dehydrogenase/Na^+^-K^+^-ATPase activity, and intracellular Ca^2+^ concentrations in the brain. Paeoniflorin can also suppress the production of pro-inflammatory mediators in the brain, such as IL-1β, TNF-α, and iNOS, which subsequently leads to a decrease in NO production, a decrease in JNK, p38, and NF-κB activity, and an increase in ERK1/2 activity. However, it remains unclear whether the neuroprotective effects of paeoniflorin could be mediated by these processes. **(B)** Paeoniflorin administration may suppress SAH-induced brain edema, blood-brain barrier impairments, neurological deficits, and walking disability by reducing reactive oxygen species, MDA, and 3-nitrotyrosine levels and increasing SOD levels and GSH-Px/catalase activities in the brain in a manner that is dependent on Nrf2 activation. However, how paeoniflorin triggers Nrf2 activation remains unclear.

Cerebral ischemia is a common pathological condition that can cause moderate to severe damage to brain function. It usually occurs in conditions such as hypo-perfusion, stroke, and anemia. Increased levels of oxidative stress and deficits in anti-oxidation are two common aspects of the pathogenesis of cerebral ischemia (Chen et al., 2020b). In a previous study, oral administration of paeoniflorin (60, 120, and 240 mg/kg, 7 days) in a rat model of cerebral ischemia induced by middle cerebral artery occlusion (MCAO) was shown to improve neurological deficits, reduce infarct volume, and decrease neuronal edema by increasing the level of superoxide dismutase (SOD) and decreasing the level of malondialdehyde (MDA) in the cortical area (Wu et al., 2020a) (Figure 2A). This ameliorates the pathological changes associated with cell apoptosis and alleviates the MCAO-induced increase in lactate dehydrogenase, Na^+^-K^+^-ATPase activity, and intracellular Ca^2+^ concentration (Wu et al., 2020a) (Figure 2A). In addition to oxidative stress, microglia-mediated neuroinflammation also plays a key role in the pathogenesis of cerebral ischemia (Jayaraj et al., 2019). Therefore, inhibition of neuroinflammation may be a potential strategy for the treatment of cerebral ischemia (Malone et al., 2019). Treatment with paeoniflorin at a dose of 2.5 or 5 mg/kg (i.p., twice daily) for 7 or 14 days (Guo et al., 2012; Zhang et al., 2015a) or administered intravenously at a dose of 15 or 20 mg/kg before blockade of blood flow (Tang et al., 2010) has been shown to ameliorate neurological deficits and neuronal apoptosis induced by MCAO (with or without reperfusion) by suppressing the production of pro-inflammatory mediators, such as interleukin-1β (IL-1β), tumor necrosis factor-α (TNF-α), and inducible nitric oxide synthase (iNOS), as well as the overactivation of astrocytes (Figure 2A). The pathologically elevated nitric oxide (NO) derived from iNOS, together with reactive oxygen species, causes oxido-nitrosative stress that leads to neurotoxic effects via mechanisms such as down-regulation of neurotrophic factor expression and monoamine levels (Kasala et al., 2014; Yang et al., 2017). Since paeoniflorin administration can inhibit both oxidative stress and neuroinflammation (Jayaraj et al., 2019; Wu et al., 2020a), NO may help to establish a causal relationship between the anti-oxidative stress and the anti-neuroinflammatory effect of paeoniflorin. However, since no direct evidence of the inhibitory effect of paeoniflorin on NO production has been provided in animal models of cerebral ischemia, this hypothesis should be solved in future studies (Figure 2A).

In addition to inducing oxido-nitrosative stress, overproduction of pro-inflammatory cytokines can also induce activation of c-Jun N-terminal kinase (JNK) and p38, inhibition of extracellular signal-regulated kinase 1/2 (ERK1/2), degradation of inhibitor kappa B-α (IκB-α), and activation of nuclear factor kappa B (NF-κB) (Neacsu et al., 2015; Biriken and Yazihan, 2021) (Figure 2A). The therapeutic effects of paeoniflorin in cerebral ischemia might be mediated by suppressing JNK/p38 activity, enhancing ERK1/2 activity, and inhibiting IκB-α and NF-κB expression in the hippocampus, cortex, and striatum, thereby reducing the Bax/Bcl-2 ratio in ischemic brain tissue (Tang et al., 2010; Guo et al., 2012; Zhang et al., 2015a; Zhang et al., 2017). JNK-dependent down-regulation of connexin 43 expression may also contribute to the neuroprotective effects of paeoniflorin in cerebral ischemia (Chu et al., 2017). However, whether the regulatory effects of paeoniflorin on JNK, p38, ERK1/2, IκB-α, NF-κB, and connexin 43 are directly related to its anti-oxidative stress effects remains unclear (Figure 2A). It is worth noting that studies *in vitro* have reported that pretreatment with paeoniflorin at a dose of 100 or 200 μM can protect cultured neurons from N-methyl-D-aspartic acid receptor (NMDA)- or TNF-α-induced cellular apoptosis, by rebalancing the expression of Bcl-2 and Bax and promoting CREB- and Ca^2+^/calmodulin-dependent protein kinase II (CaMKII) signaling (Guo et al., 2012; Zhang et al., 2017). Since excessive accumulation of glutamate and subsequent activation of NMDA receptors are central mechanisms for the induction of excitotoxicity in ischemic brain tissue (Chamorro et al., 2016; Fern and Matute, 2019), it is necessary to investigate whether the neuroprotective effects of paeoniflorin in cerebral ischemia are mediated by its inhibitory effects on glutamate receptors. Perhaps, researchers should use various molecular techniques to determine whether paeoniflorin can bind directly to glutamate receptors.

## Pharmacological effects of paeoniflorin in subarachnoid hemorrhage

SAH is a serious cerebrovascular disease with a high mortality rate. The infiltration of blood into the subarachnoid space of the brain after aneurysm rupture reduces cerebral blood flow and triggers neuronal apoptosis, partly through the induction of oxidative stress and neuroinflammation (Zhang et al., 2021a; Zhan et al., 2021). Therefore, inhibition of oxidative stress and neuroinflammation may be a potential strategy for the treatment of SAH. Treatment with paeoniflorin at a dose of 5 mg/kg after SAH (twice daily, i.p., 3 days) has been reported to inhibit SAH-induced brain edema, blood-brain barrier impairment, neurological deficits, and walking disability in rats by 1) reducing levels of reactive oxygen species, MDA, and 3-nitrotyrosine, 2) increasing the levels of SOD and the activity of glutathione peroxidase (GSH-Px) and catalase in a nuclear factor erythroid-related factor 2 (Nrf2)-dependent manner, and 3) inhibiting the production of pro-inflammatory cytokines in the cortex (Wang et al., 2020) (Figure 2B). These *in vivo* results were partially supported by the inhibitory effect of paeoniflorin (30 or 100 μM) on hematoma lysate-induced decrease in cell viability in primary cultured cortical neurons (Wang et al., 2020). Overall, these results indicate that paeoniflorin may be a potential drug for the treatment of SAH. However, because the precise molecular and signaling mechanisms underlying the neuroprotective effects of paeoniflorin in animal models of SAH are unclear, further studies should be conducted to elucidate the mechanistic aspect of paeoniflorin in SAH. For example, researchers should clarify whether there is a causal relationship between the anti-oxidative stress and anti-neuroinflammatory effects of paeoniflorin at SAH and how paeoniflorin induces Nrf2 activation: by up-regulating Nrf2 expression, inducing nuclear translocation of Nrf2, or direct binding to Nrf2 (Figure 2B)? Answering these questions may help to find new avenues for paeoniflorin research. Since excessive accumulation of glutamate is an important pathological change for the induction of neuronal damage in SAH (Sun et al., 2021b) and paeoniflorin can protect cultured neurons from glutamate-induced cellular damage through anti-oxidative stress and anti-apoptosis (Mao et al., 2010; Sun et al., 2012), researchers should also investigate whether the neuroprotective effects of paeoniflorin in animal models of SAH are mediated by inhibition of glutamate receptors.

## Pharmacological effects of paeoniflorin in pathogenesis associated with cognition, learning, and memory impairment

**FIGURE 3:**
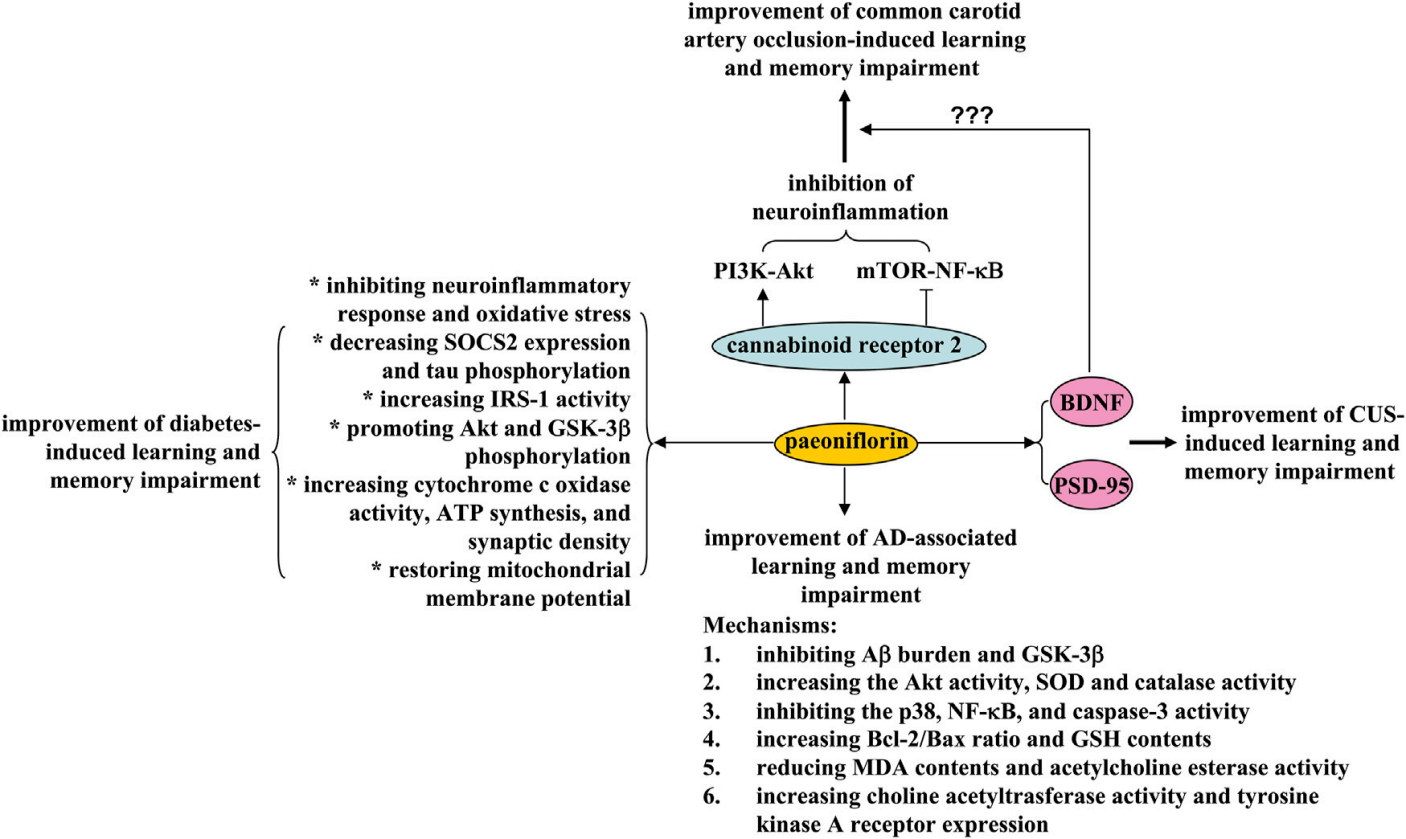
Effects and mechanisms of paeoniflorin on learning and memory impairment. Paeoniflorin administration may ameliorate 1) learning and memory impairment induced by common carotid artery occlusion by inhibiting neuroinflammation in a cannabinoid receptor 2-dependent manner; paeoniflorin-triggered activation of cannabinoid receptor 2 induces activation of PI3K-Akt signaling and inhibition of mTOR-NF-κB signaling, thereby improving learning and memory impairment induced by carotid artery occlusion, 2) diabetes-induced learning and memory impairment by inhibiting neuroinflammation and oxidative stress, decreasing SOCS2 expression and tau phosphorylation, increasing IRS-1 activity, promoting Akt and GSK-3β phosphorylation, increasing cytochrome c oxidase activity, ATP synthesis, and synaptic density, and restoration of mitochondrial membrane potential, 3) Alzheimer’s disease-associated learning and memory impairment by inhibiting Aβ burden and GSK-3β, increasing the Akt activity, SOD and catalase activity, inhibiting p38, NF-κB and caspase-3 activity, increasing Bcl-2/Bax ratio and GSH content, reducing MDA content and acetylcholine esterase activity, increasing choline acetyltrasferase activity and tyrosine kinase A receptor expression, and 4) CUS-induced learning and memory impairment by enhancing PSD-95 and BDNF expression in the brain.

Learning and memory impairment is a common phenomenon in modern society. They can be observed in various pathological conditions, such as vascular dementia, Alzheimer’s disease, and aging. Improving cognition, learning, and memory is of great importance for the treatment of neurodegenerative diseases. However, no effective drugs have been approved so far. Paeoniflorin could be a potential drug for this purpose. First, daily administration of paeoniflorin (i.p.) at a dose of 40 mg/kg over a 28-day period ameliorated the learning and memory impairment in a common carotid artery clipping model in rats by directing hippocampal microglia toward an anti-inflammatory phenotype and suppressing activation of mammalian target of rapamycin (*mTOR*)-NF-κB signaling and down-regulation of phosphoinositide 3-kinase (PI3K)-Akt signaling (Luo et al., 2018) (Figure 3). These effects of paeoniflorin may be mediated by cannabinoid receptor 2 (CB2R), as CB2R inhibition can abolish the regulatory effects of paeoniflorin on learning and memory, neuroinflammatory response, and mTOR-NF-κB and PI3K-Akt signaling (Luo et al., 2018) (Figure 3). In another study by Zhang et al. (2016), oral administration of paeoniflorin (20 and 40 mg/kg, daily for 28 days) was shown to inhibit neuronal apoptosis and increase brain BDNF expression in a rat vascular dementia model induced by bilateral common carotid artery occlusion (Zhang et al., 2016) (Figure 3). In future studies, researchers should investigate whether paeoniflorin supplementation improves learning and memory abilities in the carotid artery clipping/occlusion model by acting directly on the CB2R and whether paeoniflorin-mediated activation of CB2R improves learning and memory abilities *via* the BDNF signaling. Furthermore, as the CB2R is expressed in different cell types such as microglia (de Oliveira et al., 2022), astrocytes (Fernández-Trapero et al., 2017), neurons (Onaivi et al., 2012), and endothelial cells (Nuñez-Lumbreras et al., 2021), researchers should also determine the cellular basis for the ameliorative effects of paeoniflorin on learning and memory. Indeed, some of these hypotheses have been supported by previous studies. For example, paeoniflorin was reported to have the ability to activate the CB2R using a combination of the luc2P/CRE/Hygro reporter gene and the eukaryotic PIRES2-EGFP-CB2R expression plasmid (Luo et al., 2018).

Second, chronic treatment with paeoniflorin (5 mg/kg, i.p., 28 days) can reduce the learning and memory ability of transgenic mice with five familial Alzheimer’s disease (5× FAD) mutations via adenosine A1 receptor-dependent inhibition of Aβ burden and neuroinflammatory response (Kong et al., 2020) (Figure 3), and treatment with 2 mg/kg paeoniflorin (1 day, i.p.) can decrease escape distance and latency in mice expressing the human mutant PS2 or in mice with the genetic background C57BL/6×DBA/2 by increasing Akt activity, inhibiting p38, NF-κB, and caspase-3 activity, and increasing the Bcl-2/Bax ratio, which subsequently suppresses neuronal apoptosis and neuroinflammation in the cortex (Gu et al., 2016) (Figure 3). Moreover, chronic paeoniflorin treatment (5 mg/kg, i.p., twice daily, 4 weeks) ameliorates memory deficits in amyloid precursor protein (APP) and presenilin 1 (PS1) double-transgenic (APP/PS1) mice by inhibiting Aβ burden and neuroinflammation in a manner dependent on inhibition of glycogen synthase kinase 3β (GSK-3β) and NF-κB (Zhang et al., 2015b) (Figure 3). Further analysis showed that chronic paeoniflorin treatment (7.5, 15, and 30 mg/kg, once daily, 20 days, i.p.) can reverse Aβ_1–42_-induced decreases in GSH content and SOD and catalase activity, increases in MDA content and acetylcholine esterase activity, and decreases in choline acetyltrasferase activity and tyrosine kinase A receptor expression in rat hippocampus (Zhong et al., 2009) (Figure 3). These results indicate that anti-neuroinflammation, anti-oxidative stress, and anti-apoptosis may mediate the ameliorative effects of paeoniflorin on learning and memory deficits in animal models of Alzheimer’s disease. A puzzling question is whether and how the adenosine A1 receptor actually mediates the regulatory effects of paeoniflorin on neuroinflammation, oxidative stress, and anti-apoptosis, thereby exerting learning- and memory-enhancing effects. Is there a possibility that paeoniflorin binds directly to the adenosine A1 receptor or requires another accessory molecule to activate the adenosine A1 receptor? This question was clarified to some extent by a previous study demonstrating that paeoniflorin binds specifically with binding sites of the adenosine A1 receptor for 5′-N-ethylcarboxamido*adenosine* (*NECA*) (Liu et al., 2005b). However, it should be noted that this explanation is still a hypothesis. It should be verified by further experiments.

*In vitro* studies have reported that incubation with paeoniflorin (50, 100, and 200 μM) can ameliorate okadaic acid-induced cell swelling, microtubule de-stabilization, and tau hyper-phosphorylation in cultured SH-SY5Y cells by inhibiting the activity of calpain, GSK-3β, and Akt (Ma et al., 2018). In cultured PC12 cells, paeoniflorin (2, 10, and 50 μM) prevents Aβ_25-35_-induced cell damage by reducing cytochrome c release and caspase-3/9 activity (Li et al., 2014), and in cultured hippocampal neurons paeoniflorin can inhibit the Aβ_1–42_-induced increase in intracellular Ca^2+^, decreases in GSH contents, and increases in nitrite, carbonyl protein, and MDA levels (Zhang et al., 2015b). In addition, pretreatment with paeoniflorin (50 μM, 24 h) can prevent Aβ_1-42_-induced nuclear translocation of NF-κB and the increase in vascular endothelial growth factor (VEGF) expression levels, which in turn reduces microglial activation and migration (Liu et al., 2015). Whether the findings in cultured cells occur in Alzheimer’s disease *in vivo* should be determined in future studies.

In addition to its regulatory effects in vascular dementia and Alzheimer’s disease, paeoniflorin intake may also suppress cognition, learning, and memory impairments in other models of CNS disease. For example, administration of 20 mg/kg paeoniflorin (i.p., 30 days) was reported to reverse the decrease in time spent in the target quadrant caused by chronic unpredictable stress (CUS) by increasing the expression of postsynaptic density protein 95 (PSD95) and BDNF (Liu et al., 2019) (Figure 3). Oral administration of paeoniflorin (15 or 30 mg/kg, 7 days) can also decrease latency and increase time spent in the target quadrant in the Morris water maze test in diabetic rats induced by stimuli such as high-fat diet, high sucrose, or low dose streptozotocin, by reducing the production of IL-1β and TNF-α, decreasing the expression of suppressor of cytokine signaling 2 (SOCS2), increasing the activity of insulin receptor substrate-1 (IRS-1), promoting the phosphorylation of Akt and GSK-3β, and inhibiting the hyper-phosphorylation of tau protein in the hippocampus (Sun et al., 2017) (Figure 3). In addition, it was reported that daily treatment with paeoniflorin (10 mg/kg, i.p.) reversed streptozotocin-induced cognitive deficits by increasing cytochrome c oxidase activity, adenosine triphosphate (ATP) synthesis and synaptic density, restoring mitochondrial membrane potential, and inhibiting oxidative stress in the hippocampus and cortex of mice (Wang et al., 2018b), and oral administration of paeoniflorin (1 mg/kg) 90 min before testing attenuates scopolamine-induced deficits in radial maze performance in rats by increasing acetylcholine content or activating the α1-or β1-adrenergic receptor in the striatum (Ohta et al., 1993a; Ohta et al., 1993b; Ohta et al., 1993c) (Figure 3). Investigators should further determine the common molecular basis for the anti-dementia and anti-Alzheimer effects of paeoniflorin, and investigate whether the initial inhibition of Aβ deposition mediates the regulatory effects of paeoniflorin on cognition, learning, and memory in animal models of Alzheimer’s disease?

## Pharmacological effects of paeoniflorin in Parkinson’s disease

**FIGURE 4:**
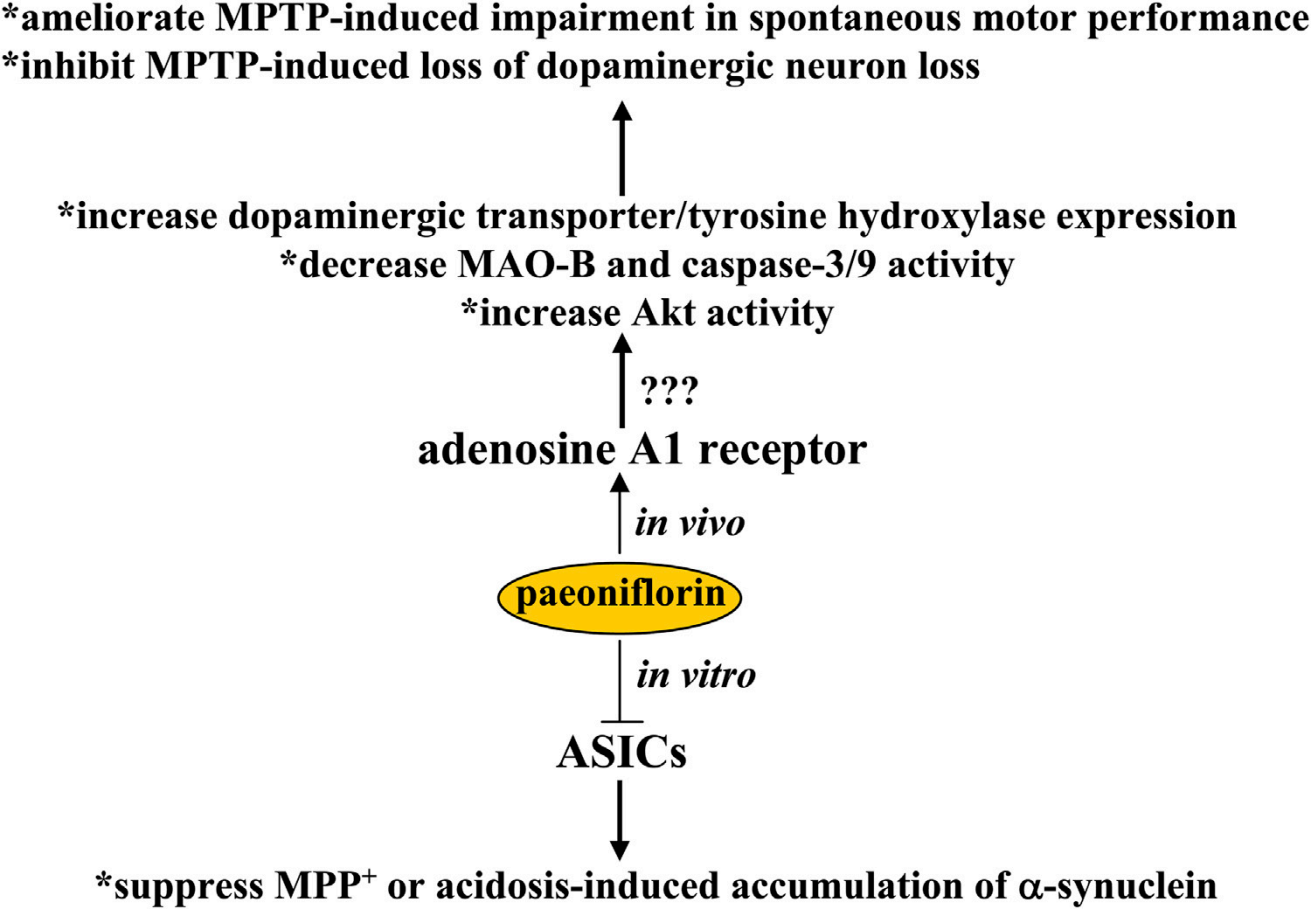
Effects and mechanisms of paeoniflorin in Parkinson’s disease. Paeoniflorin administration may ameliorate MPTP-induced impairment of spontaneous motor performance and inhibit MPTP-induced loss of dopaminergic neuron loss by increasing the expression of dopaminergic transporters and tyrosine hydroxylases, decreasing MAO-B and caspase-3/9 activity, or increasing Akt activity via a possible mechanism related to adenosine A1 receptor activation. Under *in vitro* conditions, paeoniflorin was found to suppress MPP^+^ or acidosis-induced accumulation of α-synuclein in cultured PC12 cells by inhibiting the activity or expression of ASICs. However, whether this *in vitro* effect on ASICs could mediate the neuroprotective effect of paeoniflorin in animal models of Parkinson’s disease remains unclear.

Parkinson’s disease is a devastating neurodegenerative disease in which there is progressive degeneration of striatal dopaminergic neurons. This disease can lead to severe motor disabilities that can affect the quality of daily life of patients. Due to the lack of effective drugs for the treatment of Parkinson’s disease in the clinic, this disease is in the public focus. In a previous study, oral treatment with paeoniflorin at a dose of 15 or 30 mg/kg was shown to ameliorate MPTP-induced impairments in spontaneous motor performance on the rotarod test, as well as loss of dopaminergic neurons in mice, by increasing the expression of dopaminergic transporters and tyrosine hydroxylase proteins, decreasing monoamine oxidase-B (MAO-B) and caspase-3/9 activity, and increasing Akt activity in striatal and substantia nigra (Zheng et al., 2017) (Figure 4). In another Parkinson’s disease model in rats induced by unilateral striatal 6-hydroxydopamine lesion, subcutaneous administration of paeoniflorin (5 and 10 mg/kg, twice daily for 11 days) has been shown to reduce apomorphine-induced rotation by protecting tyrosine hydroxylase-positive neurons from damage (Liu et al., 2007). In a post-treatment regimen (administered 1 h after the last MPTP dose and followed by 3 days of daily treatment at doses of 2.5 and 5 mg/kg), paeoniflorin was found to attenuate damage of to dopaminergic neurons via adenosine A1 receptor-dependent suppression of the neuroinflammatory response in the striatum (Liu et al., 2006) (Figure 4). Similar to the models of Alzheimer’s disease, further studies should clarify whether and how the adenosine A1 receptor actually mediates the regulatory effects on paeoniflorin dopaminergic cell survival and disease symptoms in animal models of Parkinson’s disease (Figure 4).

*In vitro* studies have reported that incubation with paeoniflorin (50 μM) can reduce MPP^+^ or acidosis-induced accumulation of α-synuclein and upregulate the expression of an autophagy-associated protein LC3-II in PC12 cells (Sun et al., 2011) (Figure 4), suggesting that induction of autophagy and inhibition of α-synuclein-associated pathology may mediate the anti-Parkinson’s disease effect of paeoniflorin. Incubation with paeoniflorin (50 μM) can also suppress the activity and expression of acid sensing ion channels (ASICs) in PC12 cells (Sun et al., 2011). Since the ASIC blocker amiloride can protect substantia nigra neurons from MPTP-induced neurodegeneration (Arias et al., 2008) and parkin-mediated enhancement of ASIC activities play a key role in the pathogenesis of neurodegeneration (Joch et al., 2007), the findings on the inhibition of ASICs by paeoniflorin may reveal a new mechanism for the anti-Parkinson’s effect of paeoniflorin (Figure 4). From the above results, the regulation of ASICs by incubation with paeoniflorin may be particularly interesting because ASICs have been widely reported to mediate the pathogenesis of various neuropsychiatric diseases, such as epilepsy (Biagini et al., 2001), Alzheimer’s disease (Gonzales and Sumien, 2017), and pain (Holton et al., 2020). Therefore, future studies need to investigate whether paeoniflorin can bind directly to ASICs, which could drive the development of regulators of ASICs.

## Effect of paeoniflorin on epilepsy

**FIGURE 5:**
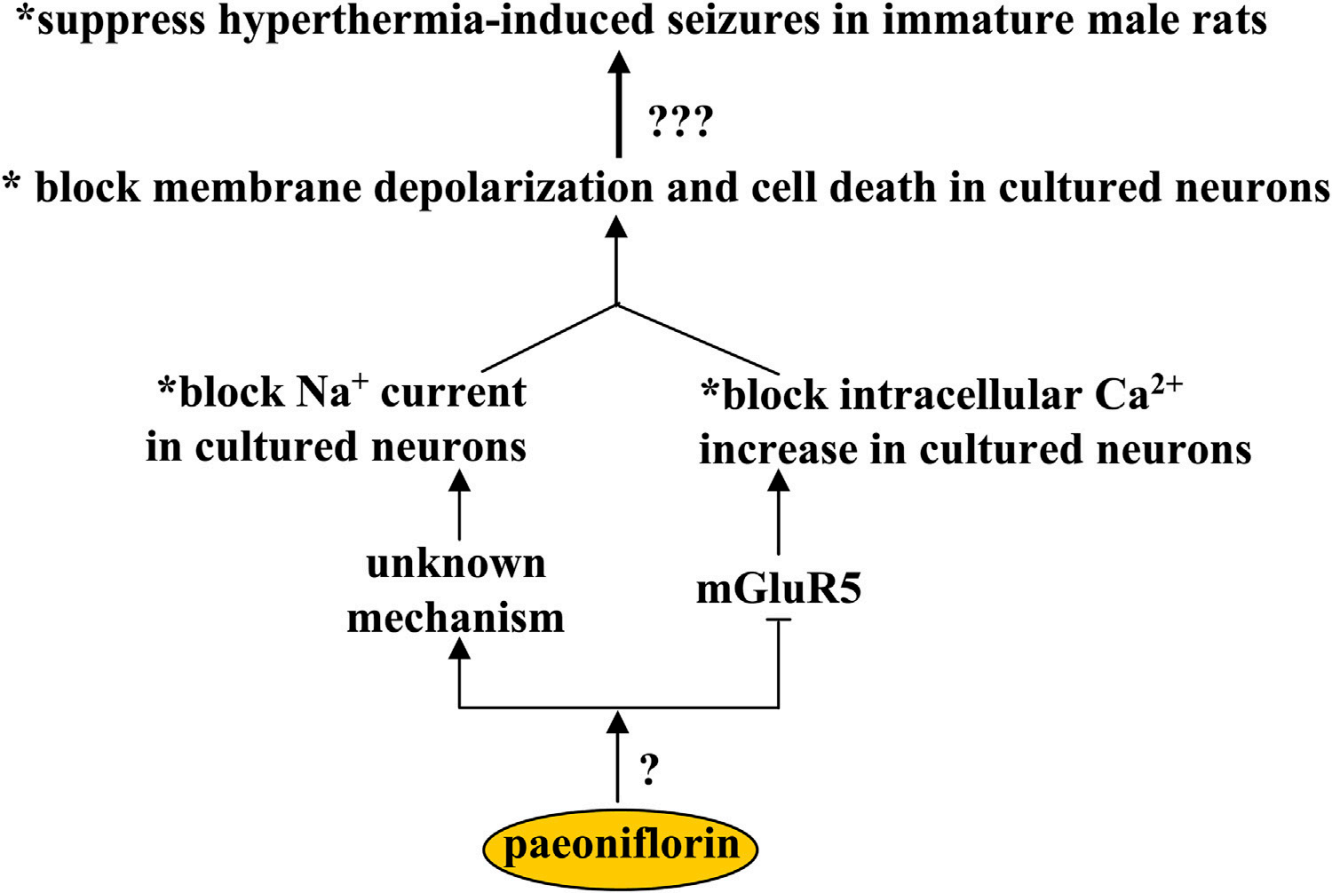
Effects and mechanisms of paeoniflorin in epilepsy. Paeoniflorin administration can suppress hyperthermia-induced seizures in immature male rats, probably by blocking membrane depolarization and cell death in primary cultured neurons. This effect of paeoniflorin on membrane depolarization and neuronal survival may be mediated by an unknown mechanism based on blocking Na^+^ current or mGluR5 inhibition that blocks intracellular Ca^2+^ increase. How paeoniflorin inhibits mGluR5 remains unclear.

Febrile seizure is the most common convulsive disorder in children (Hauser, 1994). There is a lack of effective strategies to treat febrile seizure. One potential strategy to treat febrile seizure is to correct the imbalance between glutamatergic and GABAergic neurotransmission. Paeoniflorin may be a hopeful candidate for this purpose, as oral paeoniflorin supplementation (100 mg/kg, 10 days) can suppress hyperthermia-induced seizures in immature male rats (Hino et al., 2012) (Figure 5). One of the most important mechanisms for the development of febrile seizures is the decrease of GABAnergic signals in the brain (Rating et al., 1983; Audenaert et al., 2006), and the GABA agonist can suppress hyperthermia-induced seizures in developing rats, while inhibition of the GABA receptor produces an opposite effect (Fukuda et al., 1997), suggesting that the anti-epileptic effect of paeoniflorin may be mediated by the changes in the GABAnergic signals. However, the fact is that incubation with paeoniflorin does not affect the production or release of GABA in cultured hippocampal neurons (Hino et al., 2012), which rules out the involvement of GABAnergic signals in the anti-epilepsy effect of paeoniflorin. In addition to GABA, intracellular Ca^2+^- and Na^+^-mediated *excitotoxicity* may also be involved in the anti-epilepsy effect of paeoniflorin, as the alteration of intracellular Ca^2+^ and Na^+^ levels may be closely related to the pathogenesis of epilepsy (Missiaen et al., 2000; Das et al., 2010), and incubation with paeoniflorin (50–200 or 300 μM) can block Na^+^ current (Zhang et al., 2003) and glutamate-induced increase in intracellular Ca^2+^ levels, membrane depolarization, and neuronal death in primary cultured hippocampal neurons (Hino et al., 2012) (Figure 5). Glutamate mainly acts on membrane receptors in neurons, such as the NMDA receptor, AMPA receptor, and metabolic glutamate receptors. However, the suppression of intracellular Ca^2+^ increase in cultured hippocampal neurons mediated by paeoniflorin may be mediated only by its inhibitory effects on metabolic glutamate receptor 5 (mGluR5), since incubation with paeoniflorin incubation can suppress the intracellular Ca^2+^ increase induced by an mGluR5 agonist but not by an NMDA or AMPA receptor agonist (Hino et al., 2012) (Figure 5). This finding suggests that paeoniflorin may be a negative regulator of mGluR5. However, in the absence of evidence to explain the regulatory mode of paeoniflorin on mGluR5, future studies should clarify whether paeoniflorin binds directly to the mGluR5 or requires another accessory molecule to regulate mGluR5. Future studies should also identify how paeoniflorin blocks the Na^+^ currents in primary cultured neurons. Furthermore, considering that the pathogenesis of epilepsy is related to a variety of pathological mechanisms such as astrogliosis (Seifert et al., 2010), JNK overactivation (Small et al., 2022), and microglial overactivation (Devinsky et al., 2013), researchers should conduct further experiments to investigate whether paeoniflorin exerts an antiepileptic effect via these mechanisms.

## Pharmacological effects of paeoniflorin in depression

**FIGURE 6:**
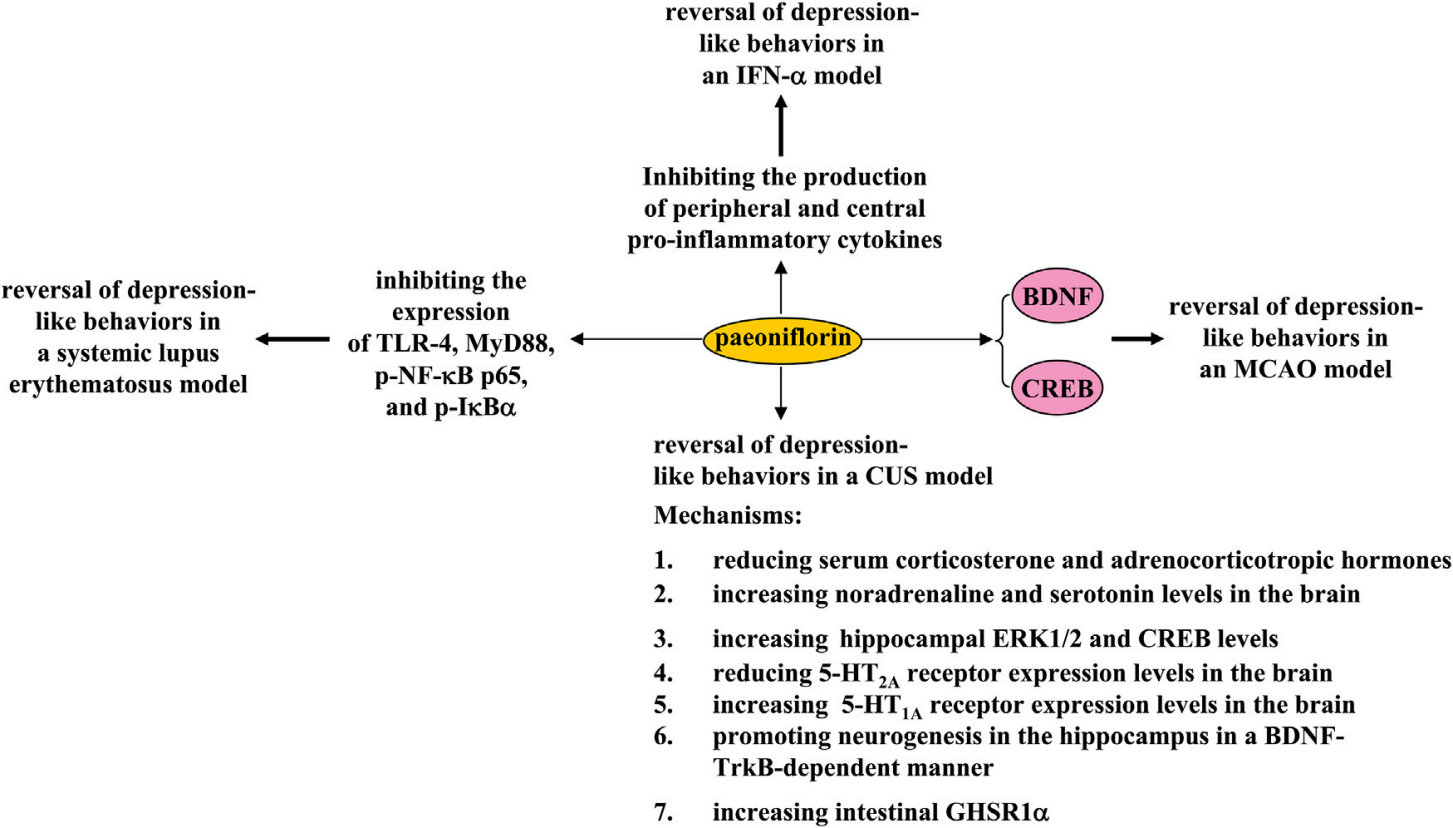
Effects and mechanisms of paeoniflorin in depression. Paeoniflorin administration can reverse 1) CUS-induced depression-like behaviors by reducing serum corticosterone and adrenocorticotropic hormone levels, increasing brain noradrenaline and serotonin levels, increasing hippocampal ERK1/2 and CREB levels, reducing 5-HT_2A_ receptor expression levels in the brain, increasing brain 5-HT_1A_ receptor expression levels, promoting hippocampal neurogenesis in a BDNF-TrkB-dependent manner, or increasing intestinal GHSR1a; 2) MCAO-induced depression-like behaviors by enhancing CREB-BDNF signaling; 3) systemic lupus erythematosus-induced depression-like behaviors by inhibiting the expression of TLR-4, MyD88, p-NF-κB p65, and p-IκBα; and 4) IFN-α-induced depression-like behaviors by inhibiting the production of peripheral and central pro-inflammatory cytokines.

Depression is a common disorder of the nervous system caused by various factors such as environmental stress and genetic mutations. Conventional antidepressants such as the selective serotonin reuptake inhibitors were developed based on the monoamine hypothesis. These drugs have a relatively good therapeutic effect in some patients but also elicit adverse effects in others (Kutzer et al., 2020; Ramic et al., 2020). Therefore, the search for new antidepressants is an urgent task for scientists. In a previous study, 30-day treatment with paeoniflorin (20 mg/kg, i.p.) was shown to reverse CUS-induced depression-like behaviors in mice by increasing BDNF and postsynaptic density protein 95 (PSD-95) expression and dendritic spine density (Liu et al., 2019) (Figure 6). Treatment with paeoniflorin at a dose of 30 or 60 mg/kg (i.p., 5 weeks, once daily) or 10 mg/kg (oral administration, 2 weeks) can also reverse CUS-induced depression-like behaviors in rats, by reducing serum corticosterone and adrenocorticotropic hormone levels and 5-HT_2A_ receptor expression levels in the hippocampus and increasing brain noradrenaline and serotonin levels, as well as hippocampal expression levels of ERK1/2, CREB, and the 5-HT_1A_ receptor (Qiu et al., 2013; Huang et al., 2015; Zhong et al., 2019) (Figure 6). The changes in ERK1/2 activities appear to be a key mechanism for the regulation of depression by paeoniflorin, as ERK1/2 inhibition can abolish the antidepressant effect of paeoniflorin (Zhong et al., 2019). The antidepressant-like effect of paeoniflorin might also be mediated by neurogenesis in the hippocampus, as paeoniflorin treatment (60 mg/kg, i.p., 4 weeks) can promote neural stem cell proliferation and increase the number of BrdU^+^ cells in the hippocampus of CUS rats by activating the BDNF-tyrosine kinase receptor B (TrkB) pathway (Chen et al., 2019) (Figure 6). This speculation may be supported by another *in vitro* discovery: paeoniflorin pretreatment (200, 400, or 800 μg/ml) can reverse hydrogen peroxide-induced apoptosis through activation of PI3K-Akt signaling in cultured neuronal progenitor cells (Wu et al., 2013).

The authors also found that paeoniflorin treatment (1 mg/kg, i.p.) produced rapid and sustained antidepressant-like effects in several models of depression in mice, including the forced swimming test and exposure to CUS, which appear to be dependent on increased expression of the gut growth hormone secretagogue receptor 1α (GHSR1α) (Zhang et al., 2021b). A major focus of this finding is the rapid and sustained antidepressant-like effect of paeoniflorin. It is well known that ketamine is an antidepressant with rapid and sustained antidepressant effects (Zanos and Gould, 2018). Although it has been reported that ketamine exerts rapid and sustained antidepressant effects *via* various mechanisms such as promoting neurotransmission and enhancing BDNF-TrkB signaling (Nguyen et al., 2016; Abdallah et al., 2018), there are still some puzzling questions about the antidepressant effects of ketamine (Price and Mathew, 2015). The intestinal GHSR1α-dependent rapid and sustained antidepressant effects provide further opportunities to investigate the antidepressant mechanisms of ketamine in detail. Given the possible similarities in the rapid and sustained antidepressant effects of paeoniflorin and ketamine, it is also necessary to investigate whether the rapid and sustained antidepressant effects of paeoniflorin are indeed mediated by BDNF-TrkB signaling.

In addition to its role in CUS models, paeoniflorin treatment may also produce antidepressant effects in some other models associated with depression. For example, paeoniflorin administration (5 mg/kg, 3 weeks, i.p.) was reported to reverse MCAO-induced depression-like behaviors in rats by enhancing CREB-BDNF signaling in the hippocampus (Hu et al., 2019) (Figure 6), suggesting that paeoniflorin administration may be a novel strategy for the treatment of post-stroke depression. In a mouse model of systemic lupus erythematosus associated with depressive symptoms, intravenous infusion of paeoniflorin (20 mg/kg, 6 h) can inhibit despair-like behaviors by reducing the expression of toll-like receptor-4 (TLR-4), myeloid differentiation primary response 88 (MyD88), phospho-NF-κB p65, and phospho-IκBα, and reducing the production of pro-inflammatory cytokines (Wang et al., 2018c) (Figure 6), suggesting that paeoniflorin could be developed as a novel drug to improve depressive symptoms in patients with systemic lupus erythematosus. Moreover, 4 weeks of pretreatment with paeoniflorin (20 or 40 mg/kg) was shown to reverse IFN-α-induced depression-like behaviors in mice by down-regulating the production of pro-inflammatory cytokines in the serum, medial prefrontal cortex, hippocampus, and amygdala (Li et al., 2017) (Figure 6).

A common signaling pathway that could be regulated by paeoniflorin is that mediated by BDNF. Regardless of the type of stress to which animals are exposed, decreased BDNF may affect many functions in the brain, such as neurogenesis, neuron survival, and neurogenesis, promoting the occurrence of depression-like behaviors in stressed animals. At this stage, it is unclear how paeoniflorin restores impaired BDNF expression and signaling under stress conditions. One possibility is that paeoniflorin enhances BDNF expression or function by activating intermittent kinases, such as ERK1/2 and CREB. Indeed, this hypothesis has been supported by numerous previously published studies. Future studies should determine how paeoniflorin triggers the activation of various intracellular kinases, such as ERK1/2 and CREB (Zhong et al., 2019). Another possibility is that paeoniflorin promotes the release of already synthesized BDNF in cell different types in the brain into their environment, and the increased extracellular BDNF acts on the TrkB receptor to mediate the antidepressant effects of paeoniflorin. These hypotheses should be investigated in future studies.

## Pharmacological effects of paeoniflorin in post traumatic stress disorder

**FIGURE 7:**
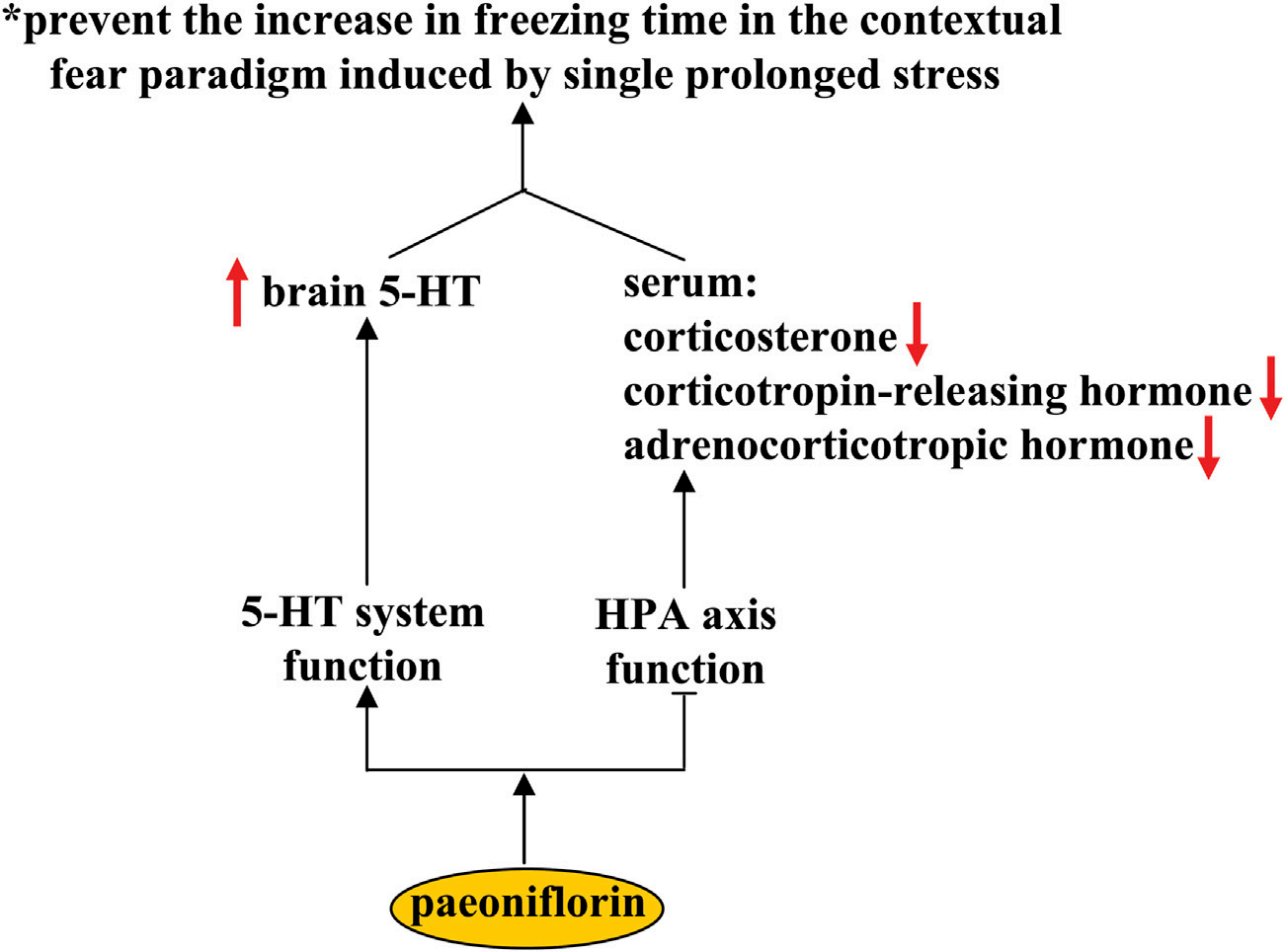
Effects and mechanisms of paeoniflorin in post-traumatic syndrome disorder. Paeoniflorin administration can prevent the increase in freezing time in the contextual fear paradigm induced by single prolonged stress by increasing brain concentrations of 5-HT and decreasing serum concentrations of corticosterone, corticotropin-releasing hormone, and adrenocorticotropic hormone. However, it is still unclear how paeoniflorin improve the function of the 5-HT system and how paeoniflorin suppresses the functions of the HPA axis.

Post traumatic stress disorder (PTSD) is a stressor- and trauma-associated disorder with a group of psychological symptoms that include re-experiencing natural disasters, anxiety, military combat, and avoidance of trauma-associated stimuli (Morland et al., 2020). Selective serotonin reuptake inhibitors are the most commonly used medications for the treatment of PTSD (Ehret, 2019). However, these medications have limited efficacy in PTSD patients. The search for new drugs is of great importance for improving the therapeutic condition of PTSD. It has been shown that pre-treatment with paeoniflorin (10 and 20 mg/kg, i.p.) can prevent the increase in freezing time in contextual fear paradigm induced by single prolonged stress (SPS) (Chen et al., 2020a) (Figure 7). Since SPS is commonly used to induce PTSD-like symptoms in rodents, these results indicate that the paeoniflorin may be a novel drug for the treatment of PTSD. Further analysis showed that pretreatment with paeoniflorin reversed the SPS-induced increases in serum corticosterone, corticotropin-releasing hormone, and adrenocorticotropic hormone, as well as the SPS-induced decrease in 5-HT levels in the prefrontal cortex and hippocampus (Chen et al., 2020a) (Figure 7), suggesting that the anti-PTSD effect of paeoniflorin may be related to its regulatory action on the HPA axis and the 5-HT system. However, more experiments should be performed in future studies before this conclusion can be drawn. In addition, because the high levels of neuroinflammatory response (Zass et al., 2017) and impairment of CREB-BDNF signaling (Ji et al., 2017) and hippocampal neurogenesis (Besnard and Sahay, 2016) have been shown to be closely related to the pathogenesis of PTSD, researchers should clarify whether the anti-PTSD effect of paeoniflorin could be mediated by its inhibitory effect on neuroinflammation and its ameliorative effect on CREB-BDNF signaling and hippocampal neurogenesis.

## Pharmacological effects of paeoniflorin in pain

**FIGURE 8:**
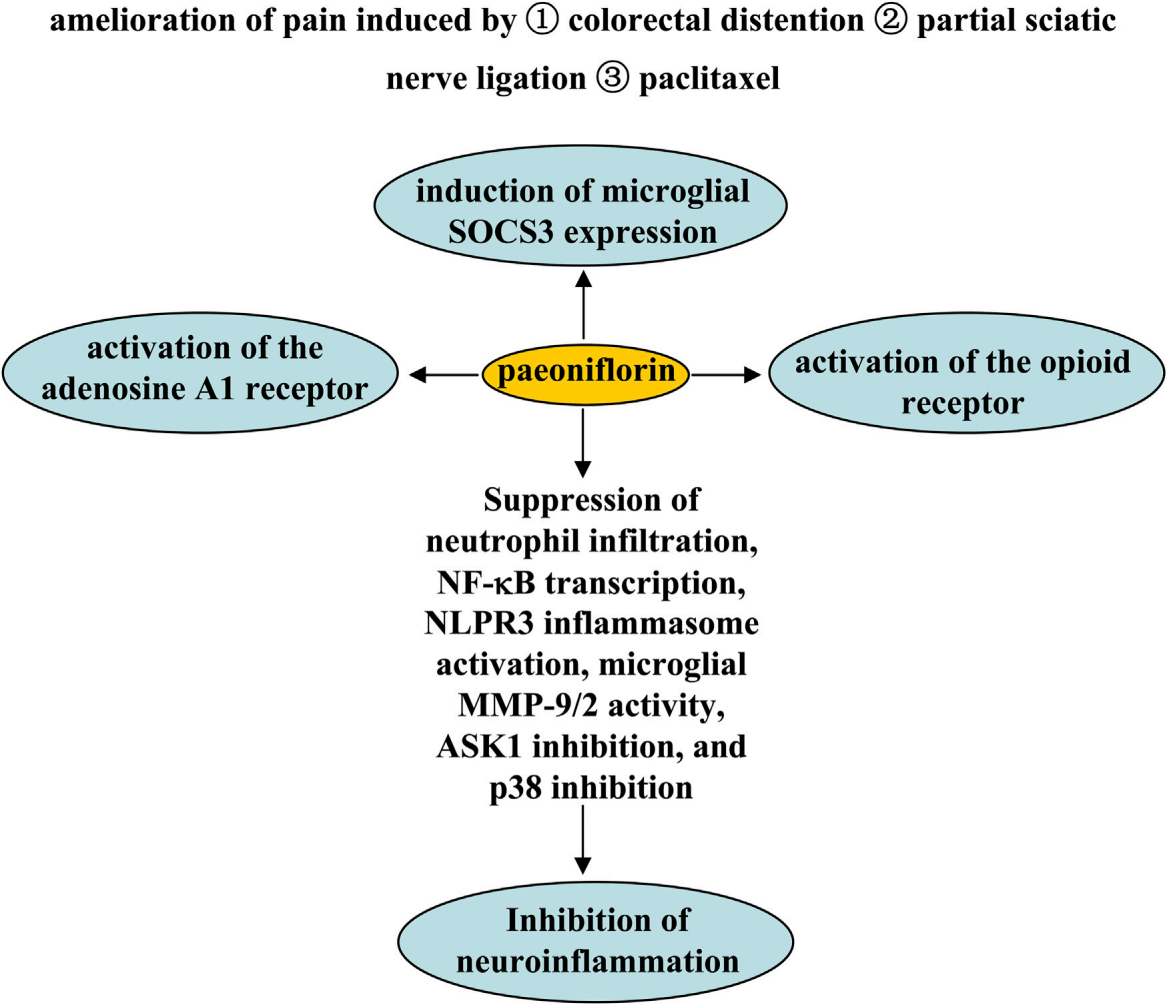
Effects and mechanisms of paeoniflorin on pain. Administration of paeoniflorin may relieve pain induced by colorectal distention partial sciatic nerve ligation, or paclitaxel. Mechanisms such as adenosine A1 and opioid receptor activation, induction of microglial SOCS3 expression, and inhibition of neutrophil infiltration, NF-κB transcription, NLPR3 inflammasome activation, microglial MMP-9/2 activity, ASK1 inhibition, and p38 inhibition may mediate the analgesic effect of paeoniflorin.

Pain is a common pathological process in various disorders such as cancer (Wu et al., 2021). The search for new mechanisms-based drugs for the treatment of pain is an old but still new focus. It has been previously reported that paeoniflorin exerts analgesic effects through a variety of mechanisms. The first mechanism is activation of the adenosine A1 receptor. Pretreatment with paeoniflorin (50 or 100 mg/kg, i.p.; 0.1 and 1% ethanol solution applied topically on plantar surfaces twice daily, beginning 24 h after model construction; 180 mg/kg, i.p.) has been shown to prevent visceral pain induced by colorectal distention in rats undergoing neonatal maternal separation (Zhang et al., 2009) and to increase mechanical threshold in mice induced by partial sciatic nerve ligation (Yin et al., 2016) or paclitaxel, an anti-microtubule agent used to treat solid neoplasms (Wu et al., 2021). Antagonization of the adenosine A1 receptor or knockout of the adenosine A1 receptor gene may abolish the analgesic effect of paeoniflorin (Zhang et al., 2009; Yin et al., 2016; Andoh et al., 2017) (Figure 8).

The second mechanism may be dependent on suppressor of cytokine signaling 3 (SOCS3). Studies by Fan et al. (2018a) showed that consecutive paeoniflorin treatment (30, 60, 90 mg/kg, i.p., 5 days) can attenuate plantar incision-induced mechanical allodynia in mice in a SOCS3-dependent manner in the spinal cord (Figure 8). *In vitro* studies have shown that incubation with paeoniflorin (0.1–10 µM) can induce SOCS3 expression in BV-2 microglia (Fan et al., 2018a), suggesting that the analgesic effect of paeoniflorin may be related to microglial SOCS3. However, since this result was obtained in microglial cell lines and there is no evidence to support the influence of microglial SOCS3 on the analgesic effect of paeoniflorin, further studies should be conducted to investigate the relationships between microglial SOCS3 and the analgesic effect of paeoniflorin. For example, researchers can use the technique of conditional SOCS3 gene knockout in microglia to confirm the influence of microglial SOCS3 on the analgesic effect of paeoniflorin.

Third, the endogenous opioid receptor may be involved in the analgesic effect of paeoniflorin, because inhibition of the opioid receptor has been shown to prevent the amelioration effect of systemic paeoniflorin administration before (5, 50, 100 mg/kg, i.p., 10 min before) or after (50 mg/kg, i.p., 5 min or 3 h after) bee venom-induced persistent spontaneous noiception, heat hypersensitivity, and paw edema and swelling in conscious rats (Yu et al., 2007) (Figure 8). It has been reported that activation of the adenosine A2a receptor can upregulate the expression of SOCS3 in lung tissue in a rat model of pulmonary hypertension (Fan et al., 2016). Future studies should investigate whether the above three mechanisms are linked in the analgesic effect of paeoniflorin.

Fourth, the anti-neuroinflammatory effect could mediate the analgesic effect of paeoniflorin. Researchers have found that treatment with paeoniflorin (50 and 100 mg/kg, i.p., 7 or 11 days) can attenuate the pain caused by chronic constriction injury by inhibiting the activation of NOD-like receptor protein 3 (NLRP3) infammasome in the spinal cord, partly by inhibiting NF-κB in the spinal cord (Liu et al., 2020). Pretreatment with paeoniflorin (5 or 25 mg/kg, i.p., 7 days; 1 or 2 μg administered intrathecally, daily, 8 days) can also prevent the inflammatory pain and paw edema induced by LPS or Freund’s complete adjuvant in mice by inhibiting neutrophil infiltration, NF-κB transcription, activation of nucleotide-binding oligomerization domain receptor protein 3 (NLPR3) inflammasome, and production of pro-inflammatory cytokines in footpad tissues (Hu et al., 2018; Yin et al., 2020) (Figure 8). In addition, administration of paeoniflorin (30, 60, and 90 mg/kg, i.p., 7 days; 40 mg/kg, i.p., 21 days; 50 mg/kg, i.p., once daily, 15 days) ameliorated plantar incision-induced mechanical allodynia in mice by inhibiting matrix metalloproteinases-*9/2* (MMP-9/2) activity in microglia (Fan et al., 2018b), or mechanical and thermal thresholds in rats, stimulated by chronic constrictive injury through apoptosis signal-regulating kinase 1 (ASK1) inhibition (Zhou et al., 2019) or p38 inhibition (Zhou et al., 2016)-dependent suppression of pro-inflammatory cytokine production in the spinal cord (Figure 8). In future studies, researchers should investigate the correlations between the anti-neuroinflammatory effects of paeoniflorin and the adenosine A2a receptor or the opioid receptor in the spinal cord. In addition, researchers should investigate whether paeoniflorin affects the adenosine A2a receptor or the opioid receptor in a manner mediated by direct binding of paeoniflorin to these receptors? Regarding the regulation of the adenosine A2a receptor by paeoniflorin, researchers might consult the study on the activation of the adenosine A1 receptor by paeoniflorin (Liu et al., 2005b).

## Pharmacological effect of paeoniflorin on glioblastoma

**FIGURE 9:**
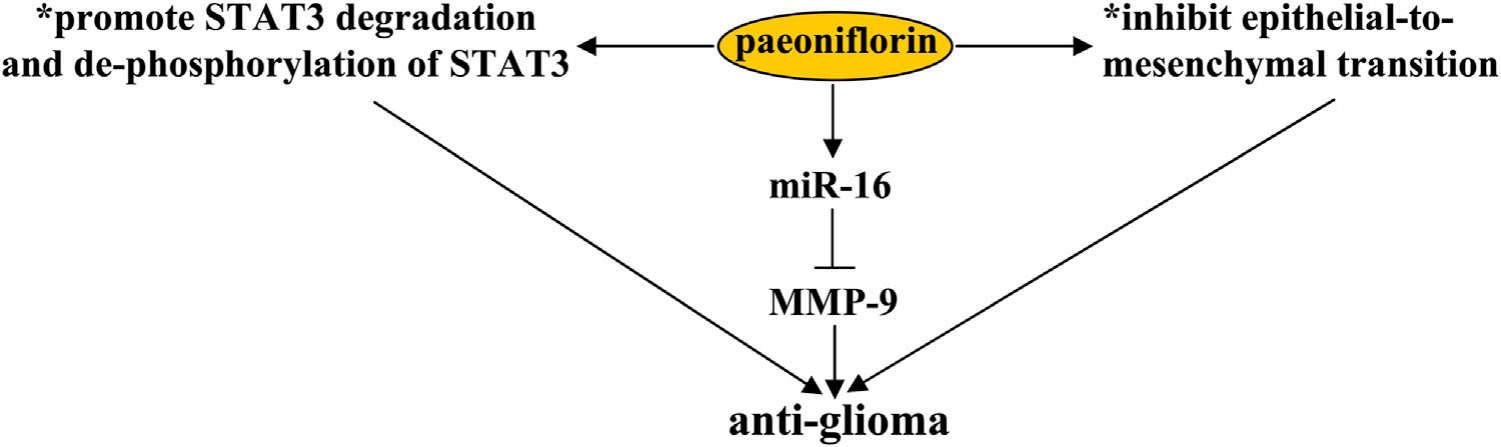
Effects and mechanisms of paeoniflorin in glioma. Paeoniflorin administration can suppress glioma growth by promoting STAT3 degradation and de-phosphorylation of STAT3 and inhibiting epithelial-to-mesenchymal transition. Increasing the expression of miR-16 is another mechanism for the anti-glioma effect of paeoniflorin. The increased miR-16 upon paeoniflorin treatment induces glioma cell apoptosis by down-regulating MMP-9 expression.

Glioblastoma (GMB) is a common brain tumor in the nervous system. In the clinic, methods available for the treatment of GMB include chemotherapy, radiotherapy, and surgery (Janjua et al., 2021). However, although these methods are useful for ameliorating GMB symptoms, the median survival time of patients receiving these therapies is usually less than 18 months. Therefore, there is an urgent need to search for new drugs for GMB treatment. C-Met-mediated ras homolog family member A (RhoA)/Rho-associated protein kinase (*ROCK*) signaling (Xiong et al., 2016) and epithelial-to-mesenchymal transition (EMT), characterized by loss of cell polarity, play important roles in tumor invasion and metastasis (Coelho et al., 2020). Their inhibition is a promising strategy for GMB treatment. Previous studies have shown that treatment with paeoniflorin (10, 15, 20, or 40 μM) suppresses hepatocyte growth factor-induced migration and invasion and actin cytoskeleton rearrangement in glioblastoma cells, by inhibiting c-Met-mediated RhoA/ROCK signaling and EMT in U87, U251, and T98G cells or in the U87 xenograft mouse model, thereby reducing tumor size (Figure 9) (Wang et al., 2018d; Yu et al., 2019a). Another study by Nie et al. (2015) had shown that paeoniflorin treatment at a dose of 10, 15, or 20 mM can increase cellular apoptosis and inhibit cellular proliferation in U87 and U251 cells by promoting the degradation and de-phosphorylation of Signal Transducer and Activator of Transcription 3 (STAT3) (Figure 9) (Nie et al., 2015). Since over-expression of STAT3 in glioma cells can induce resistance to the effects of paeoniflorin on cellular apoptosis and proliferation (Nie et al., 2015), these results indicate that STAT3 may be a key protein in the regulation of glioma cells by paeoniflorin. In future studies, researchers should investigate how exactly paeoniflorin promotes the degradation of STAT3 in glioma cells.

Studies by Li et al. (2015) had reported that the anti-glioma effect of paeoniflorin might also be related to miR-16, a unique miRNA whose level was reduced in glioma cell lines (Yang et al., 2014), because treatment with paeoniflorin (10 and 20 μM) can increase the levels of miR-16 in U87 cells and the pro-apoptotic effect of paeoniflorin can be abrogated by the anti-miR-16 antibody in cultured U87 cells (Figure 9) (Li et al., 2015). Further analysis suggested that the pro-apoptotic effect of paeoniflorin may be due to miR-16-mediated down-regulation of the expression of matrix metalloproteinase-9 (MMP-9), an indicator of malignant human brain glioma (Figure 9) (Nakano et al., 1995), as exogenous miR-16 up-regulation can reduce the expression levels of MMP-9 protein in U87 cells (Li et al., 2015). Considering the importance of miR-16 in glioma development (Li et al., 2013; Yang et al., 2014), future studies should investigate the exact mechanisms mediating the down-regulatory effect of miR-16 on MMP-9 expression.

In glioma studies, various glioblastoma cells with different gene mutation backgrounds have been established to test the anti-glioma effects of different compounds. In clinical practice, the glioblastomas of different patients also have different gene mutation background, so the currently used anticancer drugs are not as effective against glioma cells (Venkatesan et al., 2016). The search for new agents that play a comprehensive role in suppressing glioblastoma is of great importance in improving the current therapeutic situation in glioma. It is known that the lack of comprehensive therapeutic effects of anti-glioma drugs is mediated in part by O6-methylguanine-DNA methyltransferase (MGMT)-mediated drug resistance (Lang et al., 2021). The absence of MGMT protein may predict sensitivity to temozolomide (TMZ) (van Nifterik et al., 2010). Interestingly, one of the aforementioned glioblastoma cell lines T98G was reported to have high MGMT levels (van Nifterik et al., 2010; Szeliga et al., 2012), which may indicate high resistance to TMZ. Since the addition of paeoniflorin can strongly suppress the growth of T98G cells (Wang et al., 2018d), compare with TMZ, paeoniflorin may have advantages in the treatment of glioma. However, more studies should be conducted in future investigations before definitive conclusions can be drawn. Researcher should also determine whether paeoniflorin affect the sensitivity of glioma cells to TMZ by altering the expression of MGMT.

## Conclusion

In this review, the authors outline the pharmacological effects of paeoniflorin on the nervous system. Paeoniflorin may exert preventive and/or therapeutic effects on nervous system disorders through two common mechanisms: anti-oxidative stress and anti-neuroinflammation (Liu et al., 2006; Guo et al., 2012; Cong et al., 2019; Liu et al., 2020). The changes in signaling molecules such as CREB (Zhang et al., 2017; Hu et al., 2019), BDNF (Chen et al., 2019), ERK1/2 (Zhong et al., 2019), CaMKII (Zhang et al., 2017), Akt (Ma et al., 2018), GSK-3β (Ma et al., 2018), IκB-α and NF-κB (Neacsu et al., 2015; Biriken and Yazihan, 2021), JNK (Chu et al., 2017), p38 (Gu et al., 2016), and STAT3 (Nie et al., 2015) may mediate the pharmacological effects of paeoniflorin in the nervous system. In glioblastoma cells, paeoniflorin treatment can suppress tumor cell migration and invasion and actin cytoskeleton rearrangement by inhibiting c-Met-mediated RhoA/ROCK signaling and EMT (Wang et al., 2018d; Yu et al., 2019a), deactivating STAT3 signaling (Nie et al., 2015), or increasing miR-16 expression (Li et al., 2015). Activation of the adenosine A1 receptor (Liu et al., 2006; Kong et al., 2020) and opioid receptor (Yu et al., 2007), enhancement of SOCS3 signaling (Fan et al., 2018a), and inhibition of microglial activation (Liu et al., 2020) play critical roles in the analgesic effects of paeoniflorin. In general, paeoniflorin intake may be beneficial in the prevention and/or treatment of nervous system disorders, including cerebral ischemia (Guo et al., 2012; Zhang et al., 2015a; Hu et al., 2019; Wu et al., 2020a), SAH (Wang et al., 2020), vascular dementia (Zhang et al., 2016; Luo et al., 2018), Alzheimer’s disease (Gu et al., 2016; Kong et al., 2020), Parkinson’s disease (Liu et al., 2006; Liu et al., 2007; Zheng et al., 2017), depression (Qiu et al., 2013; Huang et al., 2015; Zhang et al., 2021b), PTSD (Chen et al., 2020a), pain (Yu et al., 2007; Zhang et al., 2009; Yin et al., 2016; Andoh et al., 2017; Fan et al., 2018a), epilepsy (Hino et al., 2012), and glioblastoma (Nie et al., 2015; Wang et al., 2018d; Yu et al., 2019a). Further exploration of its therapeutic basis in nervous system disorders may promote the future application and development of paeoniflorin.

Of particular note, although paeoniflorin can be administered by various routes, oral administration may result in low concentrations of paeoniflorin in the brain, which may be due to its poor oral bioavailability (Yu et al., 2019b) and may limit its use in the treatment of disease. However, it should also be noted that once paeoniflorin enters into the body, it can be converted into another molecule that crosses the blood-brain barrier, benzoic acid (Yu et al., 2019b). Benzoic acid supplementation can improve cognitive function in patients with schizophrenia (Lane et al., 2013), dementia (Lin et al., 2019; Lin et al., 2021), and early-phase Alzheimer’s disease (Lin et al., 2014), and benzoic acid derivatives have been reported to exert numerous pharmacological effects in the nervous system, such as suppression of glaucoma-associated neurodegeneration (Luo et al., 2020), inhibition of microglial activation and pathological processes of amyotrophic lateral sclerosis (Ibarburu et al., 2021), and inhibition of proliferation of human glioma cells (Yue et al., 2016), researchers should clarify whether the pharmacological effects of paeoniflorin in the central nervous system are mediated by benzoic acid.
